# Network Pharmacology-Based Dissection of the Mechanism of Drynariae Rhizoma for Low Back Pain

**DOI:** 10.1155/2022/6092424

**Published:** 2022-10-17

**Authors:** Feng Wen, Jun Yu, Yan Cheng

**Affiliations:** ^1^Hubei University of Chinese Medicine, Wuhan 430061, China; ^2^Affiliated Hospital of Hubei University of Chinese Medicine, Wuhan 430061, China; ^3^Hubei Provincial Hospital of Traditional Chinese Medicine, Wuhan 430061, China; ^4^Hubei Provincial Academy of Traditional Chinese Medicine, Wuhan 430073, China; ^5^Hubei Aerospace Hospital, Xiaogan 432000, China

## Abstract

**Objective:**

To explain the potential mechanisms of Drynariae Rhizoma (DR) in the treatment of low back pain (LBP).

**Design:**

Network pharmacology was used to reveal the potential mechanisms including collecting the active ingredients of DR, analyzing the common gene targets of LBP and DR, constructing protein-protein interaction (PPI) network, collecting protein classification, performing Gene Ontology (GO) functional analysis and Kyoto Encyclopedia of Genes and Genomes (KEGG) pathway enrichment analysis, and verifying significant gene targets.

**Results:**

234 different gene targets and 18 active compounds altogether were obtained. AKT1, VEGFA, and HIF1A were deemed to be major gene targets based on the degree values. According to GO analysis, steroid metabolic process involved 42 (18.10%) potential therapeutic LBP targets, neuronal cell body involved 24 (10.30%) potential therapeutic LBP targets, and protein serine/threonine kinase activity involved 28 (12.02%) potential therapeutic LBP targets in biological process (BP), cellular component (CC), and molecular function (MF), respectively. According to KEGG and pathway interaction analyses, the PI3K-Akt signaling pathway involved 34 (15.89%) potential therapeutic LBP targets, and PI3K-Akt signaling pathway played a significant role in the treatment of LBP. The mRNA expression levels of AKT1 and HIF1A were upregulated in healthy nucleus pulposus (NP) tissue than in degenerative NP tissue. In contrast, the mRNA expression level of VEGFA was downregulated in healthy NP tissue than in degenerative NP tissue.

**Conclusions:**

In this study, we identified a potential relationship between LBP and DR in this work, as well as a synergistic mechanism of DR in the treatment of LBP, which serves as a benchmark for further in vivo and in vitro research.

## 1. Introduction

Low back pain (LBP) is a very common and high-impact health problem all over the world. The lifetime prevalence of LBP is 84%, the prevalence of chronic LBP is approximately 23%, and 11% to 12% of people are disabled due to LBP [1, 2]. The researches have shown that low back pain (LBP) is the second most commonly diagnosed pain condition in the United States, and although most people experience pain resolution in the acute phase, an estimated 40% experience persistent pain [3, 4]. Two-thirds of adults will suffer from low back pain and its associated disorders at some point in their lives [5]. LBP is now clearly recognized as a major public health problem. Symptoms of LBP are the second most common complaint after the common cold. In 70% of cases, LBP has no apparent etiology or well-known pathogenesis [5, 6]. Conventional treatments including drug therapy and surgery have shown some effects. Likewise, these treatments also brought some adverse effects. For example, some surgical treatments experienced failure. Even worse, some people will repeat surgical interventions at a rate of 13.4% to 32% [2]. Under the circumstances, better treatments should be widely applied to clinics.

Traditional Chinese medicine (TCM) is based on the fundamental theory of balance between yin and yang and five basic elements [4]. TCM has been widely used to treat numerous diseases such as intervertebral disc degenerative diseases [7]. Acupuncture and Chinese herbal medicine are frequently used to successfully treat pain-related disorders such as neck pain and low back pain. Due to its low risk of side effects, safety, and effective results, Drynariae Rhizoma (DR), a type of Chinese herbal medicine (gusuibu), is frequently used in the treatment of osteoporosis and fracture [8, 9]. The clinical outcomes have confirmed that naringin, a main active component of the DR, can alleviate the symptoms of low back pain (LBP). What is more, basic studies have shown that naringin enhanced cell proliferation by inhibiting TNF-*α* and MMP-3 and raising the expression of collagen II and aggrecan. This substance may also reduce local inflammation, which would slow intervertebral disc degeneration. The research also indicated that naringin may become an alternative therapeutic agent for pain associated with disc degeneration such as NP and LBP [10]. With the development of network pharmacology, we believe that multiple targets of diseases can be regulated by various ingredients contained in an herb [11]. Nonetheless, no research on the mechanisms of DR in the management of LBP has been published.

Network pharmacology has played a significant role in modern TCM research, which has provided powerful theoretical evidence for the discovery of new therapeutic targets of TCM [12]. Our study will research the new therapeutic targets of DR for LBP and provide new theoretical support for DR in the treatment of LBP.

## 2. Method

### 2.1. Screening for Active Ingredients of Drynariae Rhizoma

We screened for the active ingredients of Drynariae Rhizoma by searching for the database of traditional Chinese medicine system and analyzing platforms (TCMSP) [13] on the basis of ADME (absorption, distribution, metabolism, and excretion) criterion, which includes chemicals, targets and drug-target networks, associated drug-target-disease networks, etc. According to TCMSP, oral bioavailability (OB) ≥ 30% and druglikeness (DL) ≥ 0.18 are used to assess the potential active ingredients of DR in the treatment of LBP.

### 2.2. Searching the Chemical Structure and Gene Target of Active Ingredients

We downloaded the chemical structures of the active ingredients of DR from TCMSP or PubChem [14] and stored them in mol2 format. The PubChem database is the largest collection of freely accessible chemical information in the world. The related SMILES of these potential active ingredients obtained from TCMSP and PubChem were imported into the SwissTargetPrediction database [15] to gain gene target.

### 2.3. Gene Target Prediction between Drynariae Rhizoma and Low Back Pain

There were several databases being used to search for the gene targets associated with LBP, including Genecards database [16], DisGeNET database [17], and OMIM database [18]. Whereafter, we would delete the duplicate and wrong gene targets. Last but not least, we used the Venny tool (http://bioinfogp.cnb.csic.es/tools/venny/index.html) to obtain the common gene targets of LBP and DR.

### 2.4. Building the Ingredient-Target Network of Drynariae Rhizoma

The Cytoscape software (version 3.7.1) was used to construct the ingredient-target network of DR with the utilization of the obtained common gene targets. The Cytoscape software is an open source software platform for visualizing molecular interaction networks and biological pathways and integrating these networks with annotations, gene expression profiles, and other state data [19].

### 2.5. Constructing Protein-Protein Interaction (PPI) of Drynariae Rhizoma

We used the STRING database to construct protein-protein interaction of the common gene targets of LBP and DR. The STRING database (http://string-db.org/, version 11.0) is based on completing a comprehensive and objective global network. Many available sources of protein-protein interaction information have been collected and integrated into the STRING database. The latest version (11.0) of the STRING database covers more than 5000 organisms [20]. The specific procedure was followed. Firstly, the common protein targets of LBP and DR were imported into the STRING database. Homo sapiens was selected, and the highest confidence was set 0.9 in the minimum required interaction score. Then, the TSV format of the results was exported. Meanwhile, these results were imported into the Cytoscape software (version 3.7.1) to analyze the protein-protein interaction. Finally, the network results of protein-protein interaction were exported.

### 2.6. Gene Ontology (GO) Functional Analysis and Kyoto Encyclopedia of Genes and Genomes (KEGG) Pathway Enrichment Analysis

To analyze the common gene targets of LBP and DR, we made use of Gene Ontology (GO) functional analysis and identified the important signaling pathway through Kyoto Encyclopedia of Genes and Genomes (KEGG) pathway enrichment analysis. The processes above were performed with the help of the Metascape database (http://metascape.org). Metascape is a database providing a comprehensive gene list annotation and analysis resource. The database contains functional enrichment, interactome analysis, and gene annotation [21]. The processes of analysis were followed. First, the common gene targets of NP and DR were imported into the Metascape database. Homo sapiens was chosen, and we clicked on the custom analysis. Second, the *P* value was set to 0.01, and the min enrichment was set to 5. In particular, we selected GO biological processes (BP), GO cellular components (CC), GO molecular functions (MF), and KEGG pathway. Finally, we clicked the enrichment analysis and downloaded the data of GO and KEGG pathways. The GraphPad Prism 7.0 software was used to process the data for GO analysis, and an online analysis tool (http://www.aipufu.com/index.html) was used to process the data for KEGG pathways into bubble charts.

### 2.7. Verification of the Effect of DR

We obtained nucleus pulposus (NP) tissues from two patients with low back pain. According to the Pfirrmann classification score of magnetic resonance imaging, relatively healthy NP tissue was grades I~II, and degenerated NP tissue was grades III~V [22, 23]. The Pfirrmann grades for these two NP tissues were grade II and grade IV, respectively. NP tissues were harvested under sterile conditions and immediately sent to the laboratory. Written informed consent was obtained from each patient. The study was approved by the Ethics Committee of Hubei Provincial Hospital of Traditional Chinese Medicine. The ethics number was HBZY2022-C03-02.

### 2.8. Quantitative Real-Time Polymerase Chain Reaction (qRT-PCR) Analysis

We extracted RNA from nucleus pulposus tissue as well as cells using TRIzol reagent (Ambion, Foster City, CA, USA) following the manufacturer's instructions. We apply PrimeScript RT Master Mix (Takara Bio, Shiga, Japan) to obtain first-strand cDNA of whole RNA, and for qPCR detection, we used One-Step SYBR PrimeScript RT-PCR Kit (Takara Bio). The primer sequences were designed as follows: AKT1: forward: 5′-TGGACTACCTGCACTCGGAGAA-3′, reverse: 5′-GTGCCGCAAAAGGTCTTCATGG-3′; VEGFA: forward: 5′-TTGCCTTGCTGCTCTACCTCCA-3′, reverse: 5′-GATGGCAGTAGCTGCGCTGATA-3′; H1F1A: forward: 5′-TATGAGCCAGAAGAACTTTTAGGC-3′, reverse: 5′-CACCTCTTTTGGCAAGCATCCTG-3′; and GAPDH: forward: 5′-TCCACTGGCGTCTTCACC-3′, reverse: 5′-GGCAGAGATGATGACCCTTTT-3′. We used 2^−*ΔΔ*Ct^ way to count these relative expression standards.

### 2.9. Statistical Analysis

Data are presented as mean ± standard deviation (SD). Statistical analysis was performed by the SPSS 26.0 (SPSS, Inc., Chicago, IL, USA) and GraphPad Prism 7.0 software. Each experiment was performed at least three times. Multiple group outcomes were tested by one-way analysis of variance (ANOVA) test followed by Tukey's post hoc test. The Student's *t*-test was applied to analyze the two sets of parameters. Statistical significance was *P* < 0.05.

## 3. Results

### 3.1. Active Ingredients of Drynariae Rhizoma

We obtained 71 ingredients of DR and 18 active ingredients from TCMSP. After excluding the invalid ingredients including (+)-catechin, (2R)-5,7-dihydroxy-2-(4-hydroxyphenyl)chroman-4-one,22-stigmasten-3-one, and beta-sitosterol_qt, we finally selected 18 active ingredients of DR including (2R)-5,7-dihydroxy-2-(4-hydroxyphenyl)chroman-4-one, aureusidin, eriodyctiol (flavanone), stigmasterol, beta-sitosterol, kaempferol, naringenin, (+)-catechin, eriodictyol, digallate, luteolin, 22-stigmasten-3-one, Cyclolaudenol acetate, cycloartenone, cyclolaudenol, davallioside A_qt, marioside_qt, and xanthogalenol. The basic information of 18 active ingredients is shown in Table 1.

### 3.2. Gene Target Prediction

**Figure 1:**
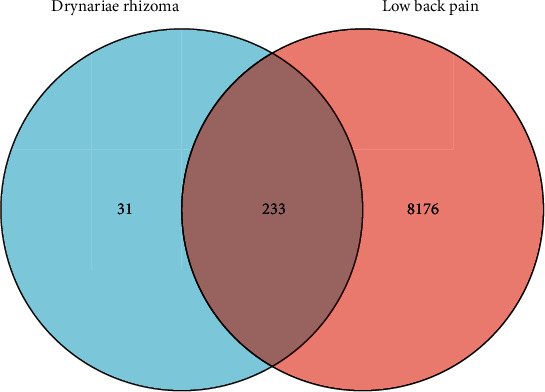
Venn diagram of common gene targets of LBP and DR.

We obtained 264 gene targets associated with DR and 8409 gene targets associated with LBP after excluding invalid and duplicate gene targets. A total of 233 common gene targets are shown in Figure 1.

### 3.3. Ingredient-Target Network of Drynariae Rhizoma

**Figure 2:**
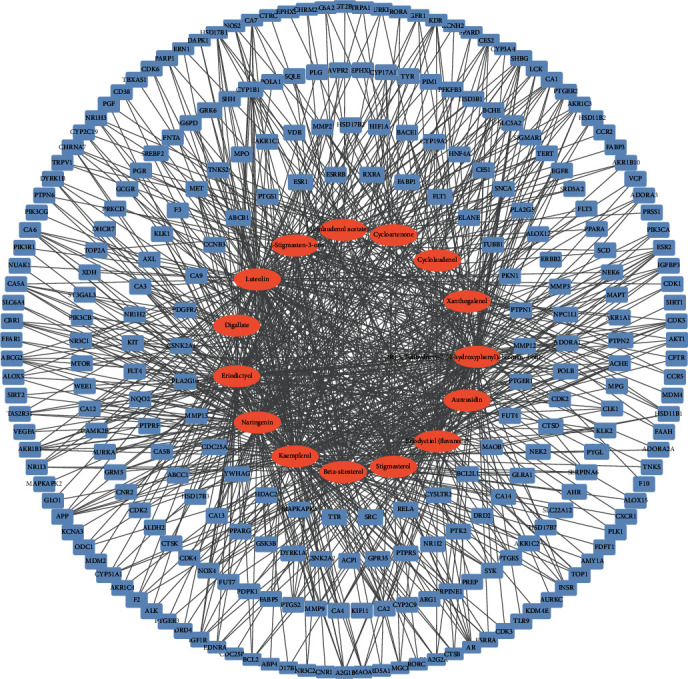
Ingredient-target network of Drynariae Rhizoma. Note: the orange oval nodes represent active ingredients of DR, and the light blue rectangle nodes are the common gene targets of LBP and DR.

The Cytoscape software was used to construct the ingredient-target network as shown in Figure 2. The orange oval nodes are 15 selected active ingredients of DR, and the light blue rectangle nodes are the common gene targets of LBP and DR. The network contains 264 nodes. According to the network, DR has multicomponent and multitarget characteristics due to the presence of multiple relationships between the active ingredient and the gene target.

### 3.4. Protein-Protein Interaction of Drynariae Rhizoma String

**Figure 3:**
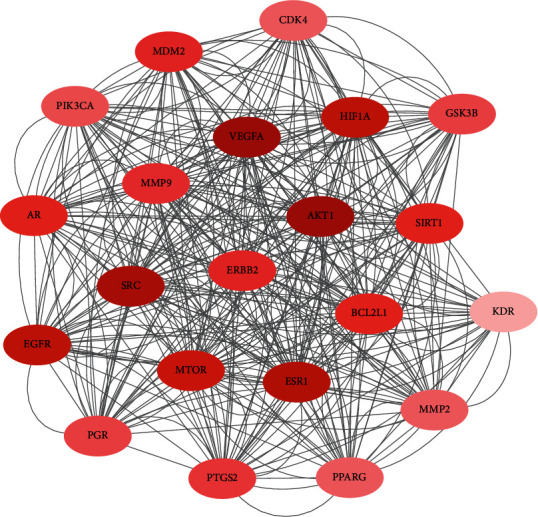
Protein-protein interaction network of Drynariae Rhizoma. Note: the size and color represent different degree values.

The network of PPI analyzed 231 common protein targets as shown in Figure 3. These nodes represent different proteins, and the size and color of these nodes represent different values of degree. The greater the value of degree, the larger these nodes and brighter the color. According to degree values, the three significant protein targets were AKT1, VEGFA, and HIF1A as shown in Table 2.

### 3.5. GO Functional Analysis and KEGG Pathway Enrichment Analysis

**Figure 4:**
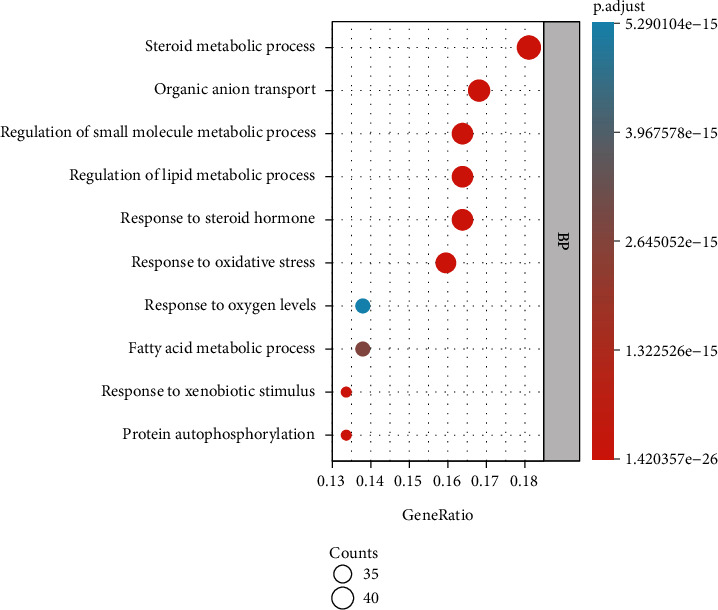
The Gene Ontology functional analysis of common gene targets.

**Figure 5:**
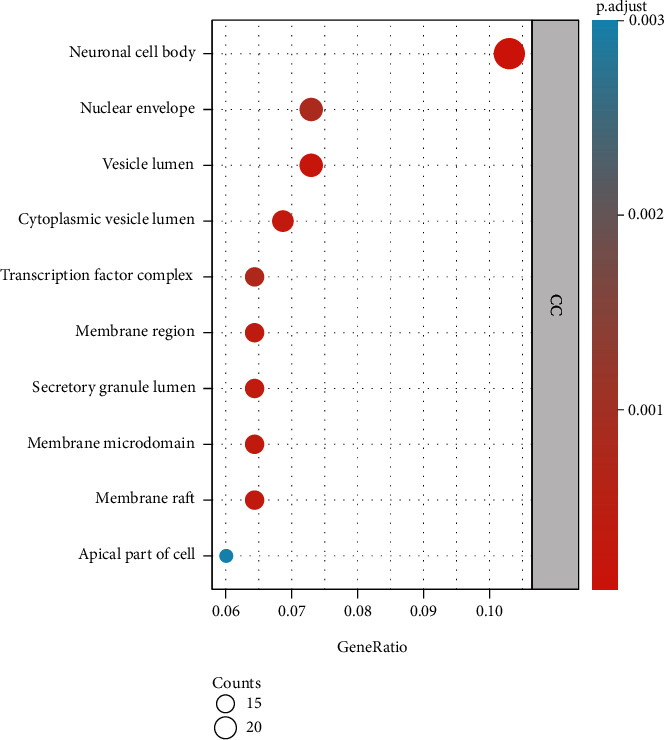
The Gene Ontology functional analysis of common gene targets.

**Figure 6:**
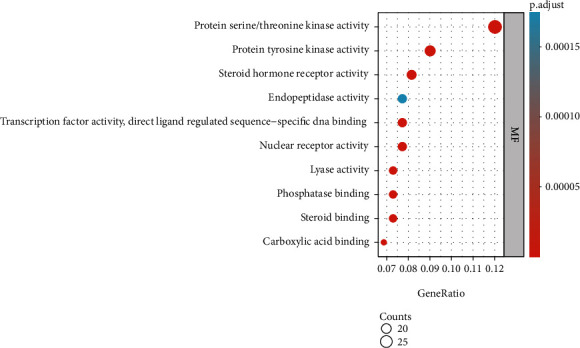
The Gene Ontology functional analysis of common gene targets.

After GO functional analysis of the common gene targets of DR and LBP through the Metascape database (*P* < 0.05), we obtained a total of 2947 enriched results, including 2478 results of biological processes (BP), 111 results of cellular components (CC), and 221 results of molecular functions (MF). We selected the top several enrichment results as shown in Figure 4. In the enrichment results of BP (Figure 4), we found that steroid metabolic process involved 42 (18.10%) potential therapeutic LBP targets, organic anion transport involved 39 (16.81%) potential therapeutic LBP targets, regulation of small molecule metabolic processes and regulation of lipid metabolic process involved 38 (16.38%) potential therapeutic LBP targets, and so on. In the enrichment results of CC (Figure 5), we found that neuronal cell body involved 24 (10.30%) potential therapeutic LBP targets, nuclear envelope and vesicle lumen involved 17 (7.30%) potential therapeutic LBP targets, cytoplasmic vesicle lumen involved 16 (6.87%) potential therapeutic LBP targets, and so on. In the enrichment results of MF (Figure 6), protein serine/threonine kinase activity involved 28 (12.02%) potential therapeutic LBP targets, protein tyrosine kinase activity involved 21 (9.01%) potential therapeutic LBP targets, steroid hormone receptor activity involved 19 (8.15%) potential therapeutic LBP targets, nuclear receptor activity and transcription factor activity, direct ligand regulated sequence-specific DNA binding and endopeptidase activity involved 18 (7.72%) potential therapeutic LBP targets and so on.

**Figure 7:**
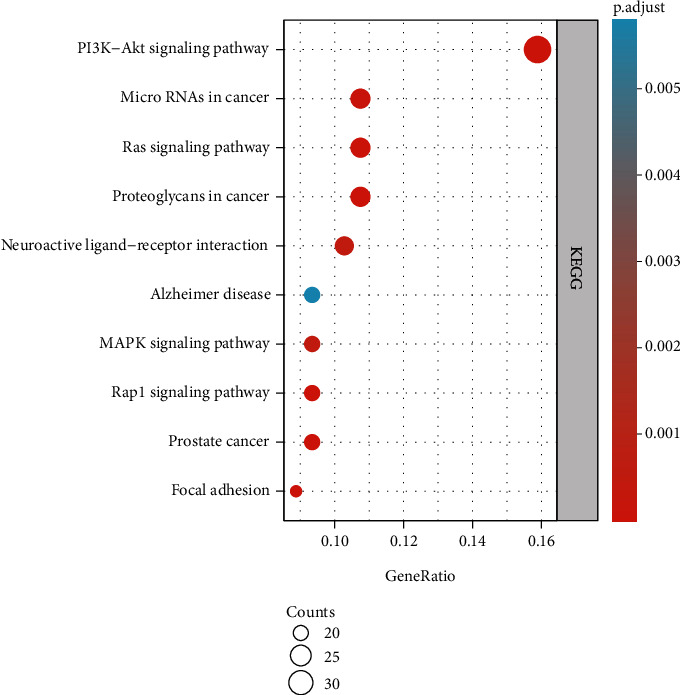
The KEGG pathway enrichment analysis of common gene targets.

After KEGG pathway enrichment analysis of the common gene targets of DR and LBP through the Metascape database (*P* < 0.05), we obtained a total of 136 enriched results. We selected the top 10 enrichment results as shown in Figure 7. According to the results of bubble chart, PI3K-Akt signaling pathway involved 34 (15.89%) potential therapeutic LBP targets, microRNAs in cancer and proteoglycans in cancer; Ras signaling pathway involved 23 (10.75%) potential therapeutic LBP targets; neuroactive ligand-receptor interaction involved 22 (10.28%) potential therapeutic LBP targets; prostate cancer, Rap1 signaling pathway, Alzheimer disease, and MAPK signaling pathway involved 20 (9.35%) potential therapeutic LBP targets and so on.

### 3.6. Validation of the Significant Protein Targets

**Figure 8:**
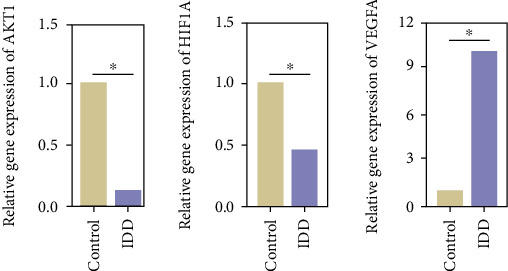
The mRNA expression levels of three significant targets.

PCR analysis was used to verify the mRNA expression levels of these 3 significant protein targets. The mRNA expression levels of AKT1 and HIF1A were upregulated in healthy NP tissue than in degenerative NP tissue. On the contrary, the mRNA expression level of VEGFA was downregulated in healthy NP tissue than in degenerative NP tissue. These results are shown in Figure 8.

## 4. Discussion

Low back pain has been a public problem, which seriously affecting people's daily life. Low back pain is mainly secondary to the diseases of cervical disc degeneration, cervical spondylosis, trauma, and so on. It is indicated that low back pain is the fourth most common reason for disability in the US, and women are more likely than men to experience low back pain [24]. We constructed a biological network between active ingredients of DR and common gene targets to reveal the mechanism of DR in the treatment of LBP. In the biological network, we selected 18 active ingredients, including (2R)-5,7-dihydroxy-2-(4-hydroxyphenyl)chroman-4-one, aureusidin, eriodyctiol (flavanone), stigmasterol, beta-sitosterol, kaempferol, naringenin, (+)-catechin, eriodictyol, digallate, luteolin, 22-stigmasten-3-one, cyclolaudenol acetate, cycloartenone, cyclolaudenol, davallioside A_qt, marioside_qt, and xanthogalenol, most of which are flavonoid compounds. As a main active ingredient, it has been reported that naringenin plays an important role in treating degenerative human nucleus pulposus cells through inhibiting the expression of inflammatory factors such as TNF-*α* [10]. Clinical evidence has revealed that non-steroidal anti-inflammatory drugs are effective for low back pain [23, 24]. Similarly, the extraction method with 70% ethanol of DR results in higher antioxidant activity [25]. Based on the strong relationship between these active ingredients and common gene targets in the network, we predict that DR will have an effect in the treatment of LBP via anti-inflammatory and antioxidant functions.

A total of 8176 gene targets of LBP were found, and a total of 264 common gene targets were selected in the network, some of which have played a significant role in the progress or cure of LBP secondary to cervical disc herniation and so on. These common gene targets have effects of anti-inflammatory, angiogenesis, proliferation, and inhibition of disc herniation, which has the similar modern drug theory of “multi-ingredients, multitarget” [26].

We constructed a PPI network to analyze the interactions of these common proteins. In this network, a total of 234 protein targets were selected. These protein targets have different effects, such as anti-inflammatory, antiapoptosis, and proliferation, some of which have been confirmed by some cell experiments. AKT1, VEGFA, and HIF1A were identified as three significant protein targets according to the degree values. Studies have shown that VEGFA plays an important role in spare nerve injury- (SNI-) induced neuropathic pain, which is mediated by enhancing the expression and colocalization of VEGFA, p-AKT, and TRPV1 in a SNI-induced neuropathic pain model, which also improves expression of VEGFA, VEGFR2, p-AKT and TRPV1 in the spinal cord [27–29]. Pasku et al and Chen et al. indicated that AKT1 was associated with disc herniation and pain. The study found that when AKT1 transcription was activated, disc herniation was deteriorated and AKT1 mRNA was related to AKT3 only in herniated discs. They also confirmed that neovascularization was associated with disc degeneration and herniation, and AKT1 was associated with angiogenesis [30, 31]. According to much evidence shown above, we predict that the 18 active ingredients of DR have the potential ability to combine with the protein targets of LBP. As a major nuclear transcription factor regulated by hypoxia, HIF-1a has a broad target gene spectrum and can regulate about 1% of all genes in human, including the following: genes related to angiogenesis, including the coding genes of VEGF and its vascular endothelial growth factor receptor (VEGFR); genes related to cell proliferation and apoptosis, including insulin-like growth factor-2 (IGF-2) and transforming growth factor-A (TGF-a) p42/p44 mitogen activated protein kinase, P13K, p53, MDM2, and other coding genes; glucose metabolism, including glucose transporters GLUT 1 and GLUT 3 and transmembrane hydrogenase; and genes related to iron metabolism, including transfer receptor and ceruloplasmin.

We performed GO functional analysis to analyze the common genes of LBP and DR. According to analysis results, we found that these common genes have multiple functions in BP, CC, and MF. In the BP, the steroid metabolic process involved 42 (18.10%) gene targets, while organic anion transport involved 39 (16.81%) gene targets, promoting cell proliferation in the treatment of LBP. A study has proposed that epidural steroid injections are one of the most common nonsurgical treatments for low back pain. In general, corticosteroid treatment often results in bone loss and osteoporosis. Neuronal cell body involved 24 (10.30%) gene targets, nuclear envelope and vesicle lumen involved 17 (7.30%) gene targets, and cytoplasmic vesicle lumen involved 16 (6.87%) gene targets in the CC, which reveals that these gene targets may make effects through plasma membrane. Protein serine/threonine kinase activity involved 28 (12.02%) gene targets, protein tyrosine kinase activity involved 21 (9.01%) gene targets, and steroid hormone receptor activity involved 19 (8.15%) gene targets in the MF, which reveals that ribonucleotide binding may play a significant role in the regulation of gene targets for DR.

We summarized the pathway enrichment analysis through the KEGG database to clarify the mechanism between DR and LBP. PI3K-Akt signaling pathway involved 34 (15.89%) gene targets, microRNAs in cancer and proteoglycans in cancer; Ras signaling pathway involved 23 (10.75%) gene targets; neuroactive ligand-receptor interaction involved 22 (10.28%) gene targets; prostate cancer, Rap1 signaling pathway, Alzheimer disease, and MAPK signaling pathway involved 20 (9.35%) gene targets and so on. In these pathways, PI3K-Akt signaling pathway and Ras signaling pathway play a significant part in the development of LBP. Xu et al. found that the activation of PI3K-Akt signaling pathway is associated with high expression of inflammatory-related factors in intervertebral disc herniation [32]. Radicular pain was contributed via the activation of p38 MAPK signaling pathway [33]. According to the network pharmacology, we provide PI3K-Akt and Ras signaling pathways as references to reveal the mechanism of DR in the treatment of LBP.

In conclusion, we used network pharmacology to indicate the potential association between DR and LBP and synergistic mechanism of DR in the treatment of LBP through prediction of gene and protein targets, which provides a reference for future study in vivo and in vitro.

## Figures and Tables

**Table 1:** Active ingredients of Drynariae Rhizoma.

Molecule ID	Molecule name	Structure	OB (%)	DL
MOL001040	(2R)-5,7-Dihydroxy-2-(4-hydroxyphenyl) chroman-4-one	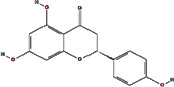	42.36	0.21
MOL001978	Aureusidin	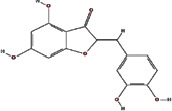	53.42	0.24
MOL002914	Eriodyctiol (flavanone)	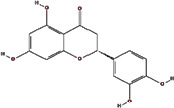	41.35	0.24
MOL000449	Stigmasterol	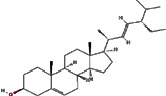	43.83	0.76
MOL000358	Beta-sitosterol	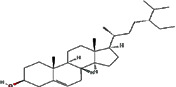	36.91	0.75
MOL000422	Kaempferol	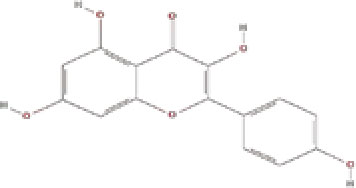	41.88	0.24
MOL004328	Naringenin	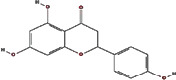	59.29	0.21
MOL005190	Eriodictyol	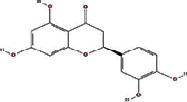	71.79	0.24
MOL000006	Luteolin	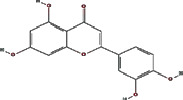	36.16	0.25
MOL009061	22-Stigmasten-3-one	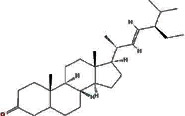	39.25	0.76
MOL009063	Cyclolaudenol acetate	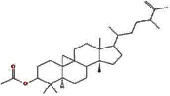	41.66	0.79
MOL009075	Cycloartenone	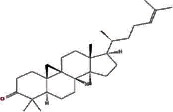	40.57	0.79
MOL000492	(+)-Catechin	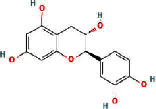	54.83	0.24
MOL000569	Digallate	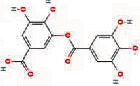	61.85	0.26
MOL009078	Davallioside A_qt	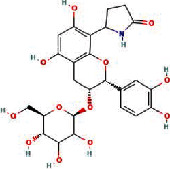	62.65	0.51
MOL009087	marioside_qt	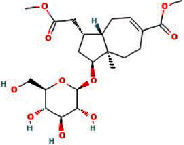	70.79	0.19
MOL009076	Cyclolaudenol	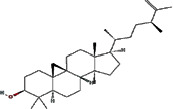	39.05	0.79
MOL009091	Xanthogalenol	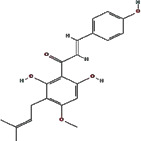	41.08	0.32

**Table 2:** Basic information of three significant protein targets.

No.	Gene targets	Degree	Betweenness centrality	Closeness centrality
1	AKT1	78	54.833	0.976
2	VEGFA	78	46.911	0.976
3	HIF1A	72	33.905	0.909

## Data Availability

Our data can be found in the TCMSP database.
